# Choices in land representation materially affect modeled biofuel carbon intensity estimates

**DOI:** 10.1016/j.jclepro.2022.131477

**Published:** 2022-05

**Authors:** Richard J. Plevin, Jason Jones, Page Kyle, Aaron W. Levy, Michael J. Shell, Daniel J. Tanner

**Affiliations:** a Consultant, Portland, OR, USA; b ICF International Inc., Fairfax, VA, USA; c Pacific Northwest National Laboratory’s Joint Global Change Research Institute, College Park, MD, USA; d US Environmental Protection Agency, Office of Transportation and Air Quality, Washington, DC, USA

**Keywords:** Biofuel, Land use change (LUC), GCAM, GTAP, Carbon intensity, Climate change mitigation

## Abstract

Estimates of biofuel carbon intensity are uncertain and depend on modeled land use change (LUC) emissions. While analysts have focused on economic and agronomic assumptions affecting the quantity of land converted, researchers have paid less attention to how models classify land into broad categories and designate some categories as ineligible for LUC. To explore the effect of these land representation attributes, we use three versions of a global human and Earth systems model, GCAM, and compute the “carbon intensity of land-use change” (CI-LUC) from increased U.S. corn ethanol production. We consider uncertainty in model parameters along with the choice of land representation and find the latter is one of the most influential parameters on estimated CI-LUC. A version of the model that protects 90% of non-commercial land reduced estimated CI-LUC by an average of 32% across Monte Carlo trials compared to our baseline model. Another version that mimics the GTAP-BIO-ADV land representation, which protects all non-commercial land, reduced CI-LUC by an average of 19%. The results of this experiment demonstrate that land representation in biofuel LUC models is an important determinant of CI-LUC.

## 1. Introduction

An increase in crop-based biofuel production requires some combination of increasing productivity of existing land (intensification), increasing planted area (extensification), and price-induced reductions in demand for crops (Searchinger et al., 2015). To quantify these effects, analysts typically rely on economic models that consider competition in agricultural, forestry, land, fuel, and other markets in response to an exogenous increase in biofuel production (e.g., Hertel et al., 2010; Laborde and Valin, 2012; Searchinger et al., 2008; Taheripour et al., 2013; Wise et al., 2015). The net effect on global GHG^1^ emissions is computed by comparing emissions from scenarios with and without the additional biofuel; the difference is considered attributable to the additional production.

Extensification, by definition, involves the conversion of land from another use or natural land cover to crop production. This form of land use change (LUC) typically results in the gain or loss of carbon held in above- and below-ground biomass and soil (Guo and Gifford, 2002). Appropriate land categorization and representation of conversions between land-use categories is essential for understanding the role of land resources in addressing climate change (IPCC, 2019). Modeling LUC emissions requires choices about how to represent the substantial heterogeneity in land cover and land use and the carbon density of soil and biomass, and these choices directly affect LUC emissions estimates.

For the purposes of this article, we focus on how land representation and parametric uncertainty affect LUC emissions. To do so, we conduct experiments using a version of GCAM, GCAM-T 2020.0 (hereafter GCAM-T), developed through modifications to GCAM v5.1.2 (Calvin, K. et al., 2019). We leave aside non-LUC climate consequences of increasing biofuel production such as changes in fertilizer use, livestock and rice production, on-farm energy consumption (USEPA, 2010), and global consumption of transportation fuels (Plevin, 2016). We focus our discussion on two aspects of land representation: land categorization and land protection.

### 1.1. Land categorization

Here we define land categorization as the process of assigning land areas to broad land-use and land-cover categories. For modeling and inventory purposes, land categories are chosen to be broad enough to classify all land areas while accommodating national differences; readily stratified by climatic or ecological zones; robust as a basis for economic modeling and emissions and removals estimation; implementable; and, complete, in that all land areas in a country may be classified by these categories.

Global models of markets and land use employ numerous simplifications necessitated by a lack of high-resolution global agronomic, economic, and ecosystem data. Some simplifications (e.g., representing the world in a few dozen aggregated geo-political regions) also address trade-offs between precision and model usability in terms of computing resource requirements. For these reasons, models and data sets represent land using a relatively small number of distinct land categories that mask real-world heterogeneity and may obscure important differences in ecological, economic, or legal features. With these simplifications in land representations, along with the gaps in understanding of human-earth system dynamics, the projections of global land use and land cover models are inevitably approximations of real-world outcomes, with substantial uncertainties (Alexander et al., 2017).

Various models have been used to estimate the LUC emissions associated with an expansion of biofuel production, e.g., GTAP-BIO (Hertel et al., 2010), GTAP-BIO-ADV (Taheripour, F. and Tyner, W.E., 2014), GTAP-Dyn (Golub et al., 2008), MIRAGE-BioF (Laborde and Valin, 2012), GLOBIOM (Valin et al., 2015), GCAM (Wise et al., 2009), EPPA (Melillo et al., 2009; Paltsev et al., 2005), ADAGE (Ross, 2009), and FAPRI-CARD (USEPA, 2010). Each of these models offers a unique representation of land categories, geopolitical regions, and crops (Table S6 in the Supporting Information, hereafter SI). These differences likely are the result of the different intended purposes of each model, which include, *inter alia*, analyzing policies related to trade, agriculture, biofuels, and climate change mitigation. Each focus results in greater detail in some areas and less detail in others. We note that many of these models derive at least part of their data from the GTAP database, including ADAGE, EPPA, GCAM, MIRAGE-BioF, and of course, the GTAP-BIO-ADV and GTAP-Dyn models.

**Fig. 1.**
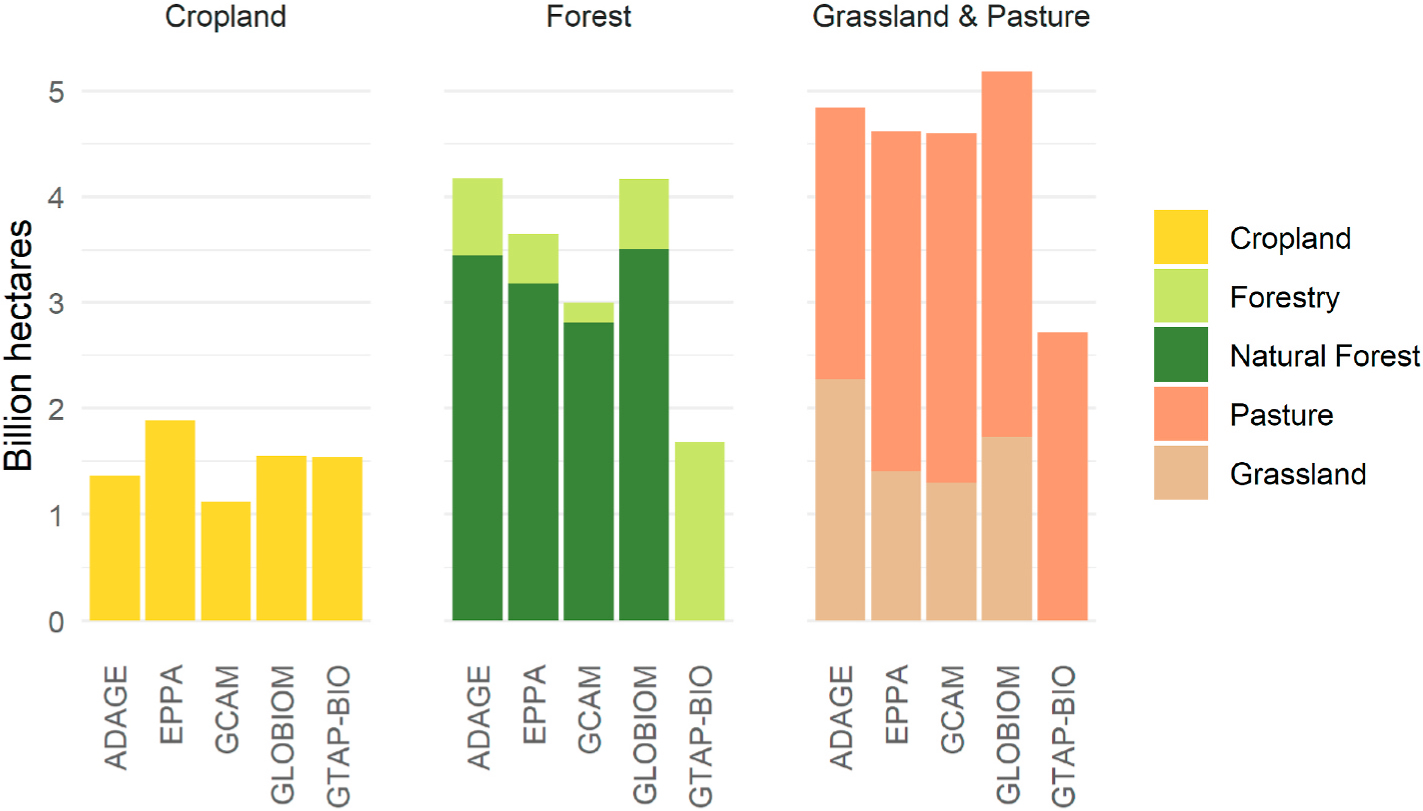
Land available for productive use in five models used to estimate biofuel-induced land use change. For simplicity, shrubland and some other land types (e.g., wetlands) are excluded.

In addition to differing categorizations of arable land, models used for biofuel-induced LUC analysis differ in the areas and types of land that are available for conversion to or from other uses. Fig. 1 shows the quantity of land in five major categories used in five models, with stacked bars indicating total forest land and total grassland/pasture. We note that the areas shown for GCAM include all non-commercial forest, grass and pasture. By default, 90% of unmanaged land is “protected” from change in GCAM, but this assumption can be altered, as we do in one of our model variants, allowing this land to be converted if market conditions so dictate. The figure shows no unmanaged land for GTAP-BIO, however, because the GTAP-BIO model equations do not consider unmanaged land, i.e., without fundamentally altering the model structure no unmanaged land is available for conversion to productive uses.

Testing the effects of all the potential models’ different land representations on CI-LUC is beyond the scope of this paper. As shown in Fig. 1, among models used for biofuel LUC analysis, GTAP-BIO has the smallest area of land available for productive use and is the only model with zero area of non-commercial land available for conversion to commercial uses. Thus, as a bounding exercise, we use GTAP-BIO’s approach to test our hypothesis that land representation choices affect CI-LUC estimates. Understanding the influence of this land representation on CI is also important given GTAP’s prominence in biofuel literature and policy. We also consider the effect of protecting different levels of non-commercial land in GCAM-T.

#### 1.1.1. GCAM land categorization

**Fig. 2.**
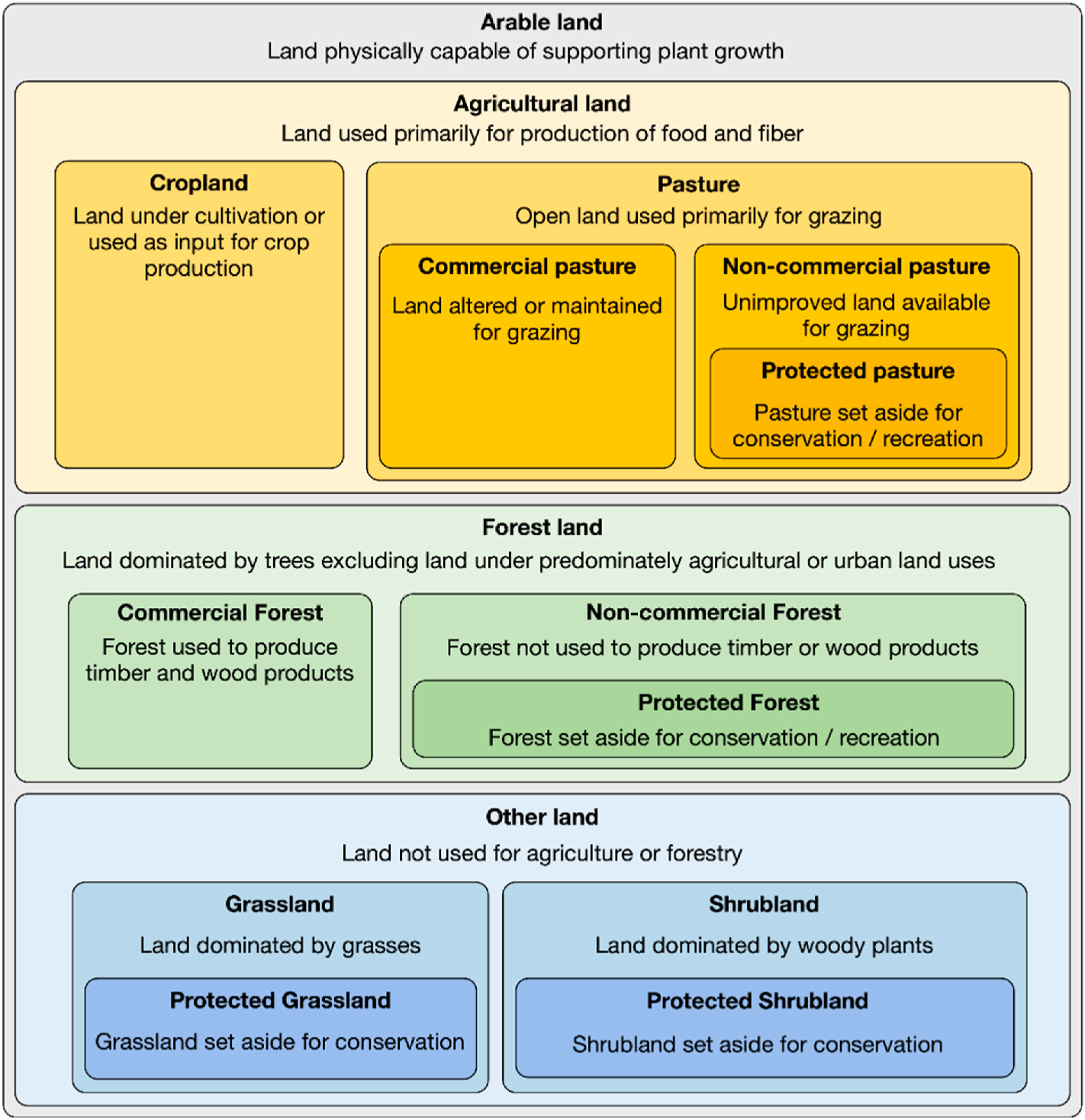
Land hierarchy in GCAM-T.

The representation of arable land in GCAM, which is unchanged in GCAM-T, is illustrated in Fig. 2. All the arable land types can transition between categories except the portion of non-commercial land that is “protected,” meaning it is removed from interacting with other land uses and thus unchanged during the modeled time horizon (see Section 1.2).

GCAM uses a land data system “to combine spatially explicit input data with tabular input data to generate spatially-referenced tabular data of crop production, crop harvested area, land value, irrigated and rainfed crop area, water footprint, soil and vegetation carbon density of unmanaged land, and historical land use/cover” (Di Vittorio, A. et al., 2020). Sources for land representation input data include maps from the History Database of the Global Environment (HYDE) (Klein Goldewijk et al., 2017) and the Center for Sustainability and the Global Environment’s (SAGE) potential vegetation dataset (Monfreda et al., 2008). HYDE estimates gridded land use for the past 12,000 years. SAGE provides natural vegetation (i.e., growth that could and would likely occur absent human interference), gridded harvest area and production between 1700 and 1992. Cropland and pasture areas are based on HYDE, and SAGE is used to classify the remaining land cover types (forest, shrubland, and grassland) (Kyle et al., 2011). The representation of crop production and land also disaggregates rainfed and irrigated agriculture based on Portmann et al. (2010).

The extent of commercial land in GCAM is calculated based on historical data on crop, livestock, and timber production. The commercial forest area in each region is calculated as the logging rate (area clear cut each year) multiplied by the rotation period (years between harvests). Thus, managed forest area represents not only the area harvested each year, but all forests that would be necessary to sustain the annual logging rate. The remaining forest in each region is categorized as non-commercial, producing neither timber nor wood products. An analogous approach is used to determine areas of commercial and non-commercial pasture by region.

One land category in GCAM that bears further mention is “non-commercial pasture,” which are lands defined as pasture in the land inventories used by GCAM (Di Vittorio, A.V. et al., 2020) but not tied to livestock commodity production in the model. Given the large quantities of land classified as pasture in some regions, particularly in comparison with the estimated feed inputs of pastoral livestock production systems, GCAM disaggregates pasture lands into “commercial” and “non-commercial” categories; the latter are treated in similar fashion to other non-commercial lands (e.g., grasslands, shrublands, forests) (Kyle et al., 2011).

#### 1.1.2. GTAP-BIO-ADV land categorization

**Fig. 3.**
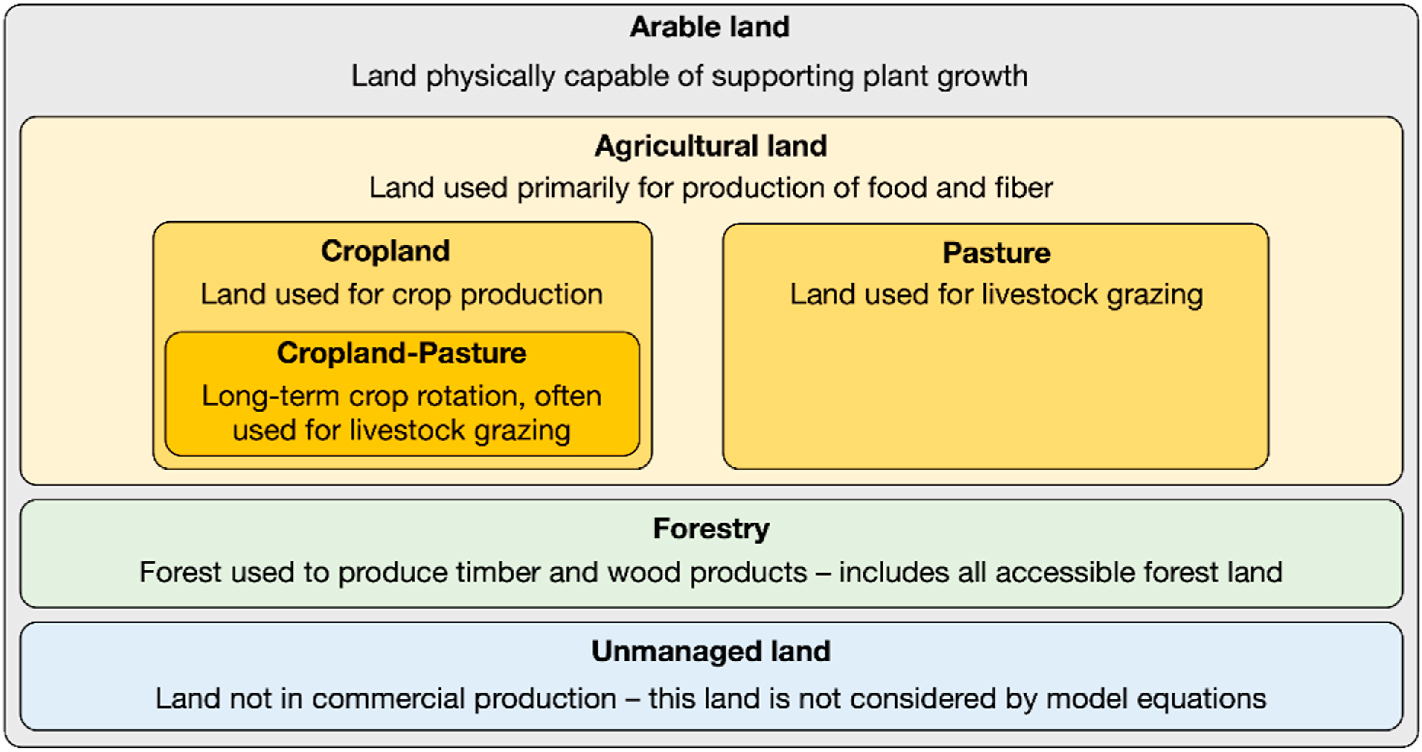
Land hierarchy in GTAP-BIO-ADV.

GTAP-BIO-ADV categorizes arable land as either cropland, pasture, forestry or unmanaged (Fig. 3.) Land categorized as cropland, pasture and forestry are modeled as commercially productive (i.e., they produce commodities), and can transition between these categories.

The GTAP land database is derived from SAGE data (Ramankutty and Foley, 1999), the UN Food and Agriculture Organization’s Global Forest Resources Assessment (FAO, 2000), and the Global Timber Model, GTM (Sohngen et al., 1999).

The GTAP land database also includes in its “forestry” category all forested land not deemed “inaccessible”—as described in Section 2.2.2—regardless of whether the land is currently in use for timber production. The baseline forestry sector output in each region is divided over this larger land area and thereafter treated as homogenous in the model. This has the effect of increasing the area of land considered to be producing timber while reducing the yield per unit area. As a result, conversion of any forested land in the model reduces timber output. An analogous treatment is applied to livestock pasture.

### 1.2. Land protection

Once land is designated into categories, models allow it to transition between categories based on economic, biophysical and other factors. Although different approaches are employed (e.g., constrained optimization, logit nesting, constant elasticity of transformation), the general logic is that land areas transition from uses with lower net returns to those with higher net returns. In addition to allowing land to transition between commercial uses, most models account for the conversion of land from non-commercial to commercial use. However, limits are generally placed on the amount of non-commercial land that can be brought into production by protecting certain areas. For example, the GLOBIOM model uses the World Database on Protected Areas to define “no expansion” areas, which are excluded in the model from conversion to agricultural or forestry activities (Valin et al., 2015).

#### 1.2.1. GCAM land protection

All recent versions of GCAM, including GCAM v5.1 (Calvin et al., 2019), assume by default that 90% by area of all non-commercial land classes (i.e., non-commercial pasture and forest, grassland and shrubland) are protected in each geographic land use region (the intersection of geopolitical regions and river basins) represented by the model. The basis for this assumption is the 2014 Energy Modeling Forum (EMF-27) model inter-comparison effort, which explored the upper limits on the quantities of land that are potentially available for conversion to bioenergy production in global long-term land use models (Popp et al., 2014).

In contrast to GCAM v5.1, the GCAM-T model (see Section 2.3) leaves unprotected all land that can be readily converted to agricultural use except for land areas designated for conservation. The areas suitable for agricultural use in GCAM-T are based on the global potential agricultural map produced by Delzeit et al. (2017), with refinements to better account for major waterbodies, urban areas, and areas that were already used for cropland. Also protected are areas listed in the World Database of Protected Areas (UNEP-WCMC and IUCN, 2018), categories I-a, I-b, and II, less previously deforested areas within the protected zones. As a result of this analysis, in total 36% of all non-commercial land is protected globally in GCAM-T (see Table S1 for protection fractions by land category and region).

#### 1.2.2. GTAP-BIO-ADV land protection

Although the GTAP land database includes a category called “unmanaged” land, this land is not referenced by the equations of the GTAP-BIO-ADV model (Taheripour and Tyner, 2009; Taheripour, F. and Tyner, W., 2014a, b). As a result, this land is effectively protected from conversion to agriculture or forestry (Avetisyan et al., 2011; Peña-Lévano et al., 2015). As discussed above, unmanaged land in the GTAP database includes all arable land that is not designated as cropland, forestry, or livestock pasture. Unmanaged land in the database includes forests deemed economically “inaccessible,” based on proximity to infrastructure, as per in the Global Forest Resources Assessment (FAO, 2000). The absence of unmanaged land from the GTAP-BIO-ADV model thereby excludes forests more than 10 km from infrastructure in most countries (Sohngen et al., 2008). It also means that all other non-commercial land types (e.g., grassland, shrubland, savanna, wetlands) are essentially protected in the model. In total, almost 2.4 billion hectares (Bha) of land are protected in this manner within the GTAP-BIO-ADV model (see Table S2 for land area by region).

### 1.3. The goal of this analysis

The goal of this study is to show how different approaches to two aspects of land representation (i.e., land categorization and land protection) affect estimates of the LUC emissions induced by increased corn ethanol production. Differences across models in the treatment of land use change dynamics and other factors have made it difficult to quantify the contribution of these land representation choices to LUC emissions. Furthermore, land categorization and protection assumptions are often embedded in the underlying databases upon which models are constructed and have thus far eluded studies of the effects of parametric uncertainty on biofuel LUC emissions (Plevin et al., 2015; Valin et al., 2015). Here, we develop different land representations within a single model, allowing us to isolate and quantify the effect of land representation on LUC emissions. In doing so, we highlight the previously understudied contribution of these land representation assumptions to uncertainty in estimates of biofuel-induced LUC emissions.

To investigate the effect of land protection on LUC emissions, we run a single model, GCAM-T, with different sets of land protection assumptions: one with 90% land protection and another with land protection assumptions based on empirical evidence. We explore the effect of land categorization by comparing these results with a third model version that mimics the GTAP-BIO-ADV approach. As discussed above, the scenario based on GTAP-BIO-ADV is useful as a bounding exercise (Fig. 1) and relevant given its prominence in biofuel literature and policy.

## 2. Methods

This study is based on a modified version of the Global Change Analysis Model (GCAM) v5.1 (Bond-Lamberty et al., 2021), developed with support from the EPA’s Office of Transportation and Air Quality, which we call GCAM-T v2020.0 (GCAM-T for simplicity). We compared this baseline version of GCAM-T with two further modified versions: one which approximates the land representation of the GTAP-BIO-ADV model, which we call “GTAP Land Proxy”, and another that modified only the land protection assumptions to use the standard GCAM approach of protecting 90% of all non-commercial land, which we call “90% Protection.” By using a single model with alternative land representations, we demonstrate the effect of these alterations on the carbon intensity of LUC induced by an increase in corn ethanol production in the U.S. (In GCAM-T, 0.0433 MJ of corn oil biodiesel is co-produced with each MJ of corn ethanol produced in the U.S., and this biodiesel is included in the denominator of the carbon intensity calculation. The shock is therefore 96% corn ethanol and 4% corn oil biodiesel. For simplicity, we refer to this experiment as a “corn ethanol” shock. We used Monte Carlo simulation (MCS) to explore the sensitivity of the model to both parametric uncertainty and to the choice of land representation. The parameter distributions and analytic methods used in the uncertainty analysis are detailed in Section S2 and Table S4.

### 2.1. Carbon intensity definition

We define carbon intensity of land use change (CI-LUC) as the projected global change in CO_2_ emissions from LUC per unit of additional corn ethanol production, with both quantities summed annually from 2021 through 2060. Emissions include only the CO_2_ from changes in above- and below-ground biomass and soil carbon. We calculate the additional biofuel production as the sum of the annualized differences in corn ethanol production in the U.S. between the scenario with increased corn ethanol production and the baseline. As GCAM-T runs on 5-year timesteps, we approximate annual additional corn ethanol production for the four years between timesteps by linear interpolation. GCAM-T endogenously annualizes LUC emissions since soil carbon emissions follow an exponential function. Although the corn ethanol shock is introduced in the 2025 time-step, we linearly interpolate the biofuel ramp-up volume from 2020, with the first change appearing in 2021. The endogenously annualized LUC emissions likewise begin in 2021. Both the changes in LUC emissions and corn ethanol increases are summed over years 2021–2060. CI-LUC is the ratio of these two sums, presented in units of g CO_2_ MJ^−1^.

### 2.2. Scenario design

With each version of the model, we created a reference scenario in which U.S. biofuel consumption volumes are set exogenously to 2015 historical levels for all modeled years after the 2010 base year. We compared this reference scenario to a scenario with an additional 5 billion gallons of corn ethanol production annually in the U.S. from 2030 through 2060, and a linear ramp-up to the additional 5 billion gallons from zero additional gallons in 2020 to 5 billion additional gallons in 2030 (see Fig. S2 for a graphical presentation of the shock). The shape of the biofuel shock was chosen to provide a stable amount of additional biofuel production over 30 years, a commonly chosen analytical period for land use change modeling in the U.S. (EPA, 2010, CARB 2009). We note that the choice of time horizon is subjective and directly affects CI estimates, with longer time horizons producing lower estimates (Plevin et al., 2010). Analysts have used different time horizons, e.g., 20 years in analyses for the E.U. (Laborde and Valin, 2012), and a compromise of 25 years in the analysis by the International Civil Aviation Organization, which included participants from the U.S. and E.U. (CORSIA, 2019). Our analysis includes 30 years of steady-state production of an extra 5 billion gallons per year, as well as 9 years of ramp-up to this steady state, during which much of the modeled LUC occurs. Fundamental differences such as these in the definition of “carbon intensity of land use change” suggests caution when comparing numerical results across models (Plevin, 2016).

### 2.3. Alternative versions of GCAM-T

As explained above, GCAM-T is a version of GCAM created through modifications to GCAM v5.1.2. We use GCAM-T as the baseline model in our experiment and create two additional versions for comparison.

#### 2.3.1. 90% protection model

This version of GCAM-T replaces the suitability-based land protection described above with the protection of 90% of all non-commercial land classes (i.e., non-commercial pasture and forest, grassland and shrubland) in each geographic land use region (the intersection of geopolitical regions and river basins) represented by the model. This is the default land protection assumption in all recent versions of GCAM other than GCAM-T.

#### 2.3.2. GTAP Land Proxy model

This version of the model uses a modified land representation inspired by the GTAP-BIO-ADV model in that it categorizes arable land as either commercially productive cropland, pasture or forestry, or as non-commercial. The GTAP Land Proxy model approximates the areal extent of commercial forest and pastureland in GTAP by modifying the GCAM data system. All non-commercial land except “other arable” land is protected from conversion to commercial production. We left “other arable” land available for conversion, similar to the special treatment of “cropland-pasture” in GTAP-BIO-ADV. To match the timber output in the GCAM-T base year, areal timber yield is adjusted downward in each region so that the product of the total combined forested land area and the lowered yield matches the base year’s commercial timber production, while the areal extent approximates the corresponding value in the GTAP database. An analogous approach is followed for livestock grazing land. The details of these changes are provided in the SI text and associated spreadsheet documents.

### 2.4. Baseline land representation in each model version

**Fig. 4.**
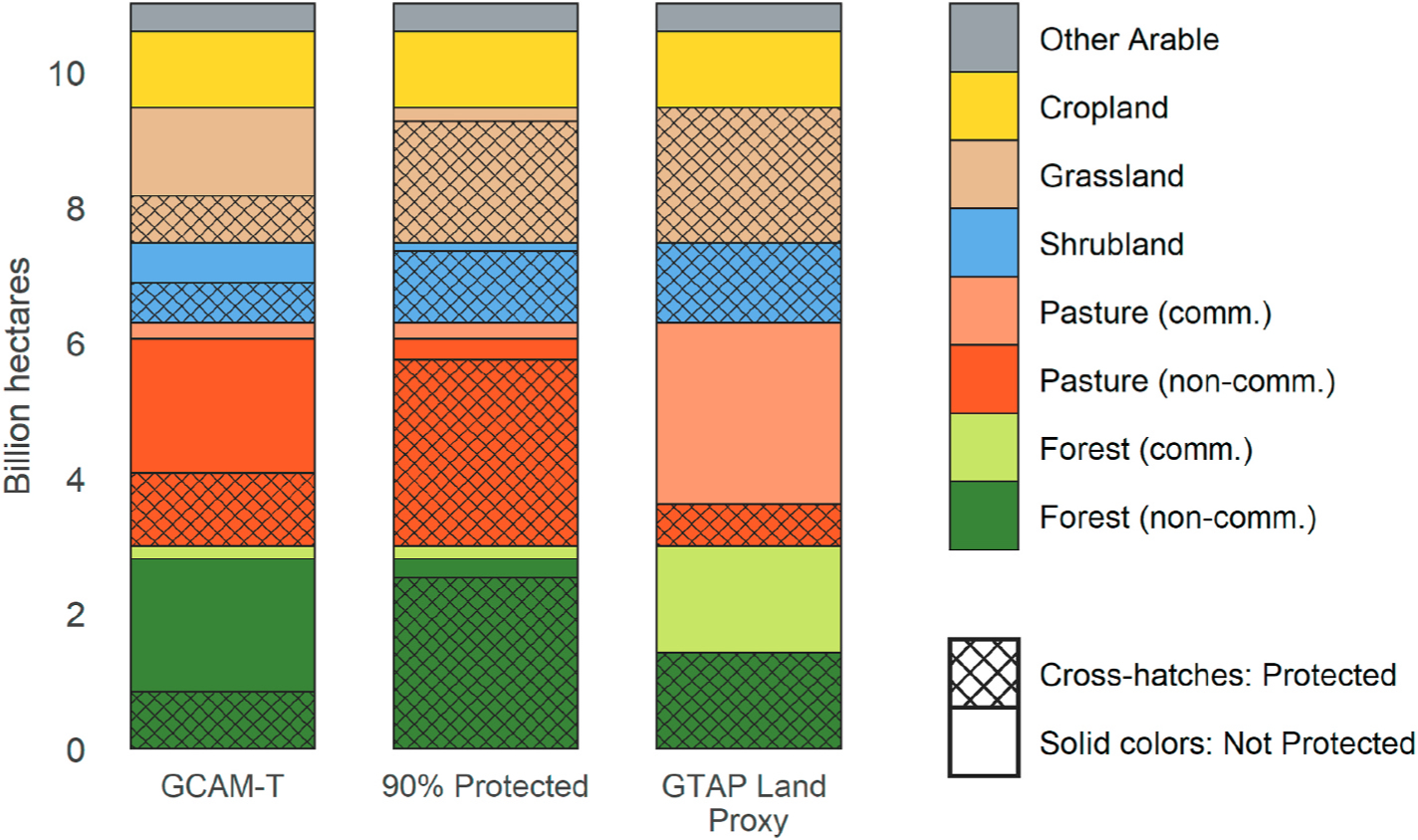
Global baseline land allocation in 2010, by land category. The cross-hatched portions of the bars represent protected lands.

The baseline land allocation in each of the three models is shown in Fig. 4. Comparing global areas among the three versions, GCAM-T has the smallest area of protected land (3.2 billion hectares, Bha) and the largest area of unprotected non-commercial land (6.2 Bha), i.e., non-commercial land that is available for conversion to grazing, crop or timber production. The 90% Protected version has the largest area of protected land (8.1 Bha) and 1.3 Bha of unprotected non-commercial land. GTAP Land Proxy has a substantially larger area of commercial land (5.4 Bha) compared to the other two versions (1.6 Bha), it protects 5.2 Bha, and it also has the smallest area of unprotected non-commercial land (0.4 Bha). The only unprotected non-commercial land in GTAP Land Proxy is in the “other arable” land category, which includes fallow land and also serves to represent differences in land area estimates between USDA and other data sources.

### 2.5. Monte Carlo Simulation

We ran a Monte Carlo simulation (MCS) by applying random values drawn from distributions to 40 parameters, i.e., sets of related values in GCAM’s input files. For the three versions of the model, we ran 1,000 trials of both the baseline and corn ethanol shock scenarios (6,000 total model runs) executed on a high-speed computing cluster. The same set of randomly drawn parameter values were used for each of the three models. For all three versions of the model, a small number of trials failed to solve; the actual number of baseline/shock pairs completed for each model version was in the 976–999 range (>97.6%). The parameter distributions used were subjective estimates of the likely range of legitimate values suggested by GCAM developers. A detailed description of parameter distributions is provided in the SI.

**Fig. 5.**
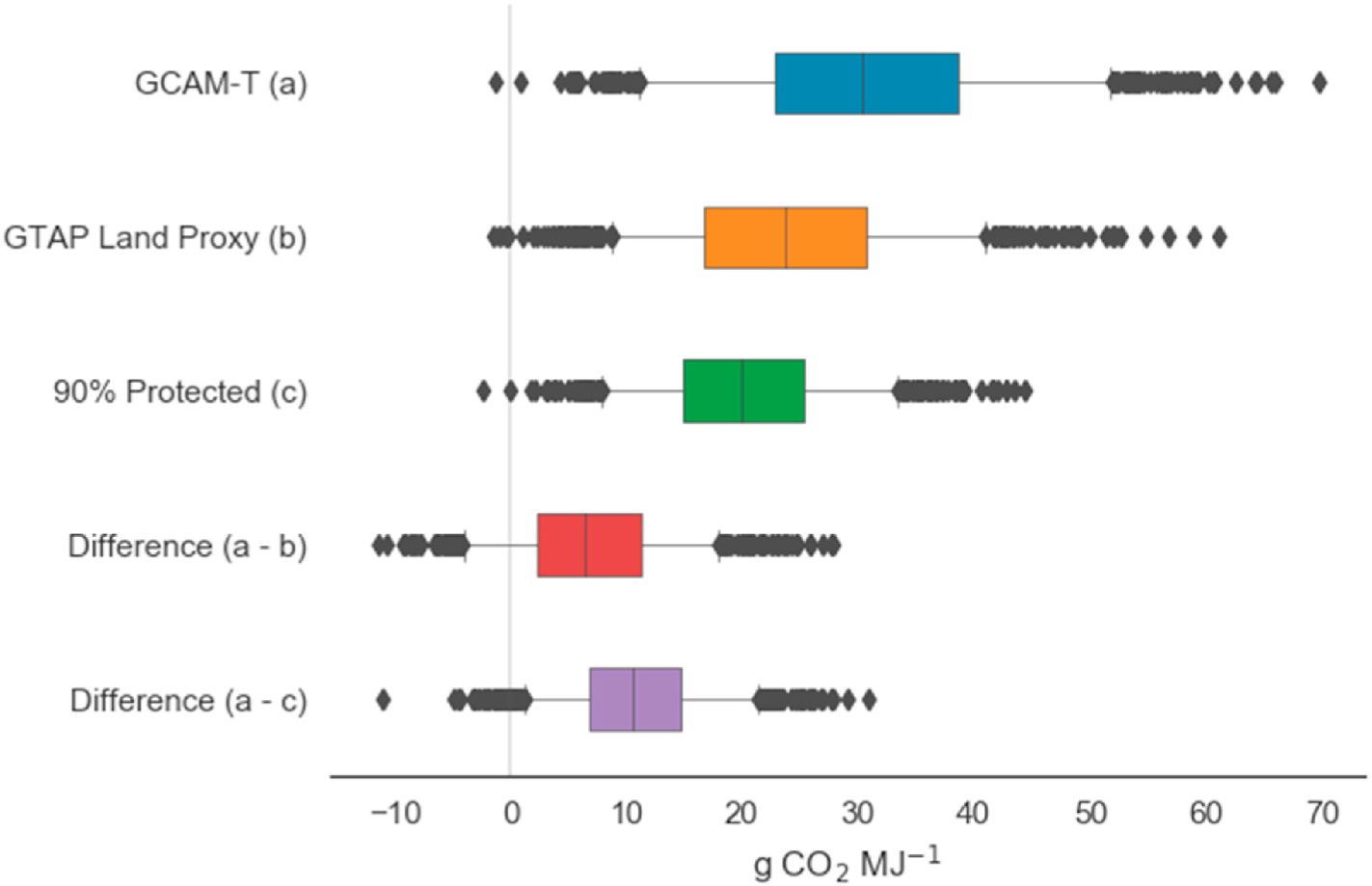
Distribution of land use change carbon intensity (CI-LUC) for corn ethanol using the three versions of GCAM-T, and distributions of the difference in CI-LUC using the same trial data in each model. Boxes indicate interquartile range; whiskers indicate 5th and 95th percentiles; vertical line indicates median value.

To consider the effect of land representation in the context of parametric uncertainty, we combined the results from the three models into a single database and created an artificial parameter, “Land Representation”, set to 0 for the GCAM-T model, 1 for the GTAP Land Proxy model, and 2 for 90% Protected model. Although “Land Representation” is a categorical value, the boxplots in Fig. 5 of per-trial differences in CI-LUC between models show that the values assigned for “Land Representation” correlate well with the prevailing results from each model. In other words, the numerical values assigned to the three model versions go from least to most restrictive in terms of land availability for crop expansion. This allows us to convert model uncertainty (approximately) into parametric uncertainty and to compare the influence of model choice to the influence of stochastic variability (See section 3.1 for a discussion of the contribution to variance of individual model parameters.).

## 3. Results

Of the trial-by-trial differences between the GCAM-T and the GTAP Land Proxy models (red box), 84% are positive, indicating that the GTAP Land Proxy model overwhelmingly results in a reduction of CI-LUC, by an average of about 7 g CO_2_e MJ^−1^ or 19%, compared to the GCAM-T model (Fig. 5). The 90% Protected model (green box) results in even greater reductions in CI-LUC compared to GCAM-T (11 g CO_2_e MJ^−1^ or 32%) (purple box.)

**Fig. 6.**
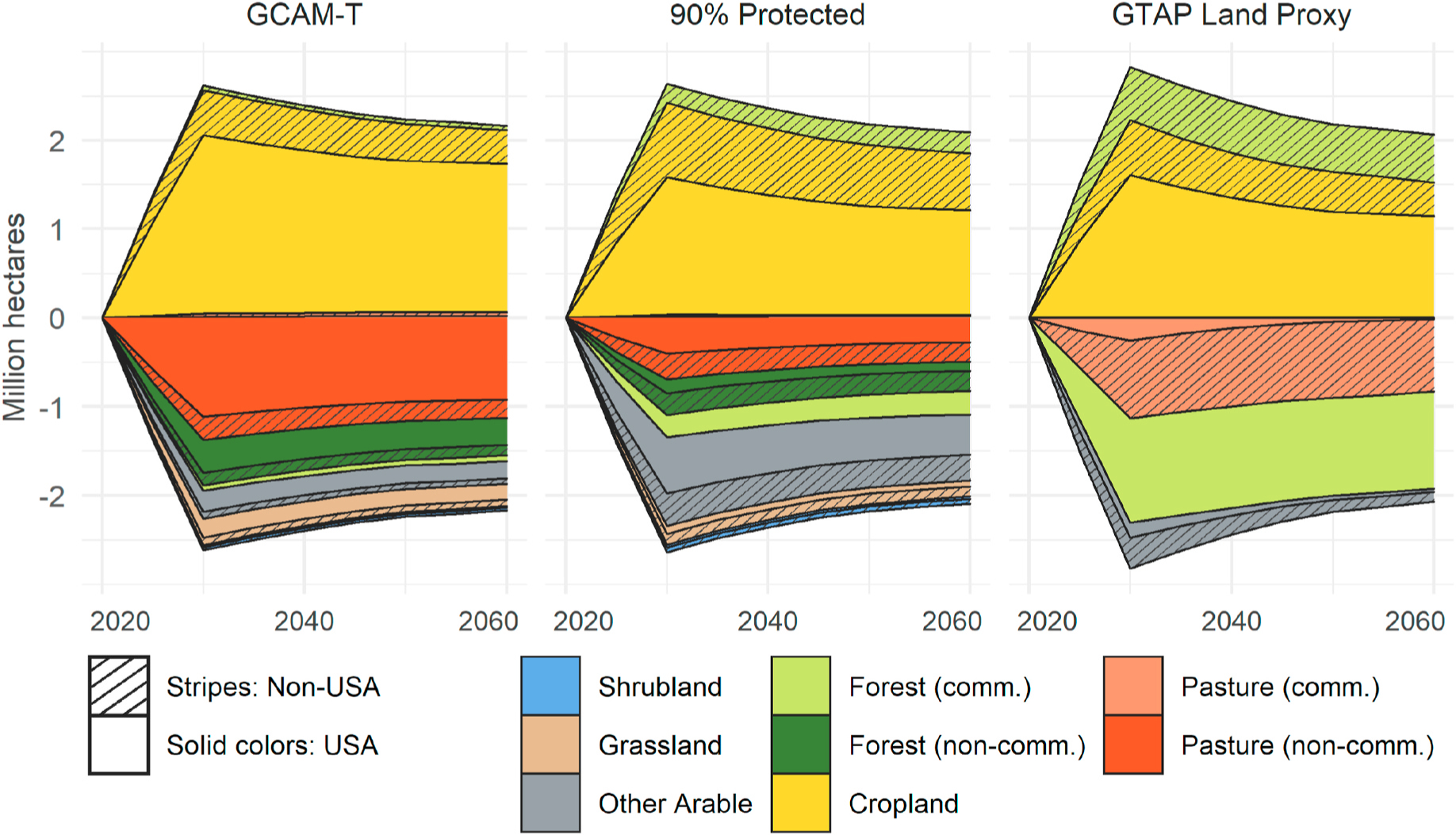
Net land use change over the modeled time horizon, in response to a corn ethanol shock starting in timestep 2025, in the three model versions: GCAM-T, 90% Protection, and GTAP Land Proxy. In the GTAP Land Proxy, the conversion of commercial forest in the USA results in afforestation in other regions in response to the reduction of timber supply lost in the USA.

Examining the non-stochastic LUC results (i.e., with default parameter values) helps to explain the differences in CI-LUC across the three versions. Fig. 6 shows the net global (USA and non-USA) changes in land in the three model versions in response to the corn ethanol shock. In all three versions, most of the LUC occurs by year 2030, the peak of the ethanol shock, and then declines as crop yields increase over time. The ethanol shock induces increased area in cropland relative to the reference scenario, though the amount of cropland expansion and the types of land affected vary by model version.

The effect of protecting land is evident from comparing the GCAM-T and 90% Protected results. The higher protection levels result in less conversion of non-commercial land and more conversion of commercial land. We see significantly more conversion of non-commercial pasture and grassland in GCAM-T and more conversion of other arable and commercial forest in 90% Protected. In 90% Protected we also see significantly more conversion of USA commercial forest, resulting in an increase in non-USA commercial forest to meet timber demands. The greater reliance on Other Arable land (a land category with relatively low carbon density) and the increase in non-USA commercial forest in 90% Protected largely explain the lower CI-LUC result compared GCAM-T.

The effect of land categorization choices is illustrated through comparison of the GTAP Land Proxy results with the two other versions. As shown in Fig. 6, there are two important differences between the models’ LUC results: (i) there is less domestic US and total expansion of cropland in the GTAP Land Proxy model, and (ii) as conversion of non-commercial land is not possible, the primary sources of new cropland are grazed pasture and commercial forest. As expected, the reduction in commercial forest – and thus, timber output – results in afforestation in other regions, as anticipated by Hertel et al. (2010). GTAP-BIO-ADV shows a similar result with a loss of forestry land in Sub-Saharan Africa and afforestation in Brazil, the rest of South America, and Russia (Fig. S3.)

In addition to differing in their estimates of land use change in response to a corn ethanol shock, the three model versions also produce significantly different projections for land use over time in the reference scenario. Unlike the other model versions, in GTAP Land Proxy the global forest area increases by approximately 0.5 billion hectares in the baseline from 2010 to 2060. As a result, in this version the corn ethanol shock induces a reduction in afforestation. Reduced afforestation produces a longer and flatter curve of LUC emissions because forest growth accumulates carbon relatively slowly over several decades. In contrast, in the other model versions the shock induces an increase in deforestation leading to a short burst of emissions that rapidly declines to zero. This is because GCAM assumes that deforestation results in instantaneous releases of biomass carbon, and soil carbon emissions that decline exponentially over time. In the GTAP Land Proxy version, annual LUC emissions in the reference scenario are approximately 4.3 Tg CO_2_ in 2060, whereas in the other model versions they are close to zero or negative by 2045. See the SI for more discussion on the differently shaped land use change emissions profiles over time.

### 3.1. Contribution to variance

We estimated contribution to variance using normalized rank (Spearman) correlations. We computed the rank correlation of the vector of values for each input parameter with the vector of values for CI-LUC, the carbon intensity of land use change. The rank correlations are squared and normalized to a percentage by dividing each by the sum of the squared correlation values. We then restore the original sign to indicate positive or negative correlation. The resulting estimates can be interpreted to represent the approximate percentage contribution of each stochastic input parameter to the variance in CI-LUC. However, the specific numerical values should not be treated as having any precise meaning, since the values are a function of which parameters were assigned distributions, and which specific distributions were assigned.

**Fig. 7.**
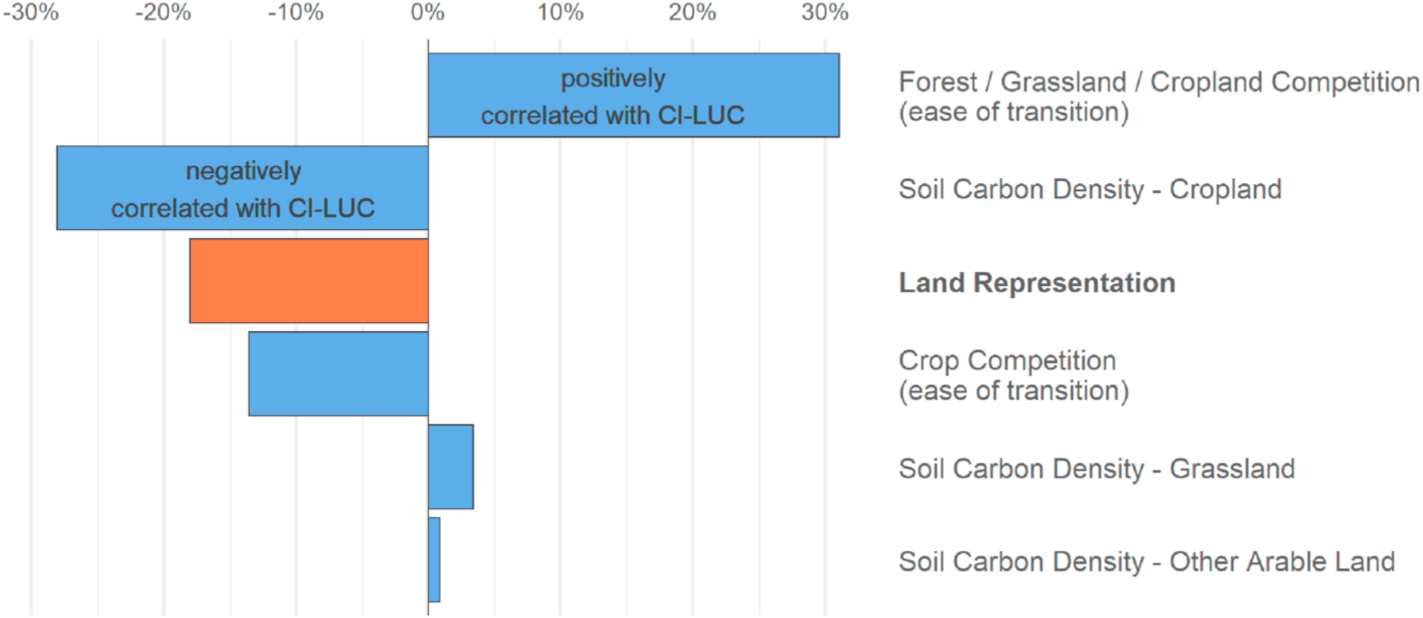
Contribution to variance of CI-LUC combining results from the three models, distinguished by a pseudo-parameter “Land Representation” (0 = GCAM-T; 1 = GTAP Land Proxy; 2 = 90% Protected). The choice of land representation is the third most influential parameter on LUC carbon intensity. See SI for parameter definitions and the implemented distributions.

Fig. 7 depicts the contribution to variance in CI-LUC of the most influential parameters that were treated as uncertain in the analysis. The first four parameters accounted for nearly all variability in CI-LUC. The most influential parameter was “Forest/Grassland/Cropland Competition”, which affects the logit exponent in the equation that controls the competition between forest, grassland, and crops. The positive value indicates that as this value increases, i.e., the easier it is to transition between these land uses, the more CI-LUC increases. Next is “Soil Carbon Density – Cropland”, a multiplier applied to all soil carbon values for cropland, globally: as this value increases, CI-LUC decreases. Since the corn ethanol shock induces an increase in cropland use, storing more carbon in these soils would result in reduced CI-LUC.

The third most influential parameter is “Land Representation.” The negative value is the result of our assignment of numerical values to the three model versions from least to most, i.e., the more restrictive the land protection, the lower the CI-LUC result.

The fourth most influential parameter was “Crop Competition”, which adjusts the competition among crops: the easier it is to switch among crops, the lower the CI-LUC result.

Based on this analysis we conclude that the top several parameters (i.e., those included in Fig. 7) materially affect estimates of corn ethanol CI-LUC. However, the specific rankings should not be treated as having any precise meaning as they are partially a function of which parameters were assigned distributions, and which specific distributions were assigned.

## 4. Discussion and conclusion

We find that the specifics of a model’s representation of land categories and of transitions among these categories strongly influence modeled biofuel CI estimates (Fig. 7). Our results highlight two ways that modeled land representation affects CI-LUC.

First, protecting more land from conversion reduces the estimated CI-LUC. Replacing the default 90% land protection assumption used in GCAM v5.1 with empirically derived estimates of available land (i.e., as in the case of GCAM-T) results in higher CI-LUC estimates. However, the protection value does not greatly influence CI-LUC until it is high enough—around 70–80%—to restrict the area available for transition. This is consistent with the findings of Calvin et al. (2014).

The second avenue through which modeled land representation affects CI-LUC is the categorization of land, as illustrated by the GTAP Land Proxy model. The GTAP Land Proxy Model protects 5.2 billion ha of non-commercial land, which translates to 58% of the non-commercial land in GCAM-T. This level of land protection, by itself, would have a relatively minor effect on CI-LUC (see Fig. S9.) Thus, we find that land categorization choices in GTAP Land Proxy are the primary reason that it produces lower estimates of CI-LUC. Eliminating all non-commercial land classes forces land conversion to occur on economically productive land. This, in turn, causes a reduction in the supply of the goods previously produced on this land, inducing price increases that trigger increased production elsewhere. In this model, conversion of forest land induces afforestation elsewhere, and this increased carbon uptake off-sets carbon emissions. The opportunity cost of converting commercially productive land requires higher prices than would be required to convert non-commercial land, resulting in some demand destruction.

Here we summarize our findings regarding the bases for the land categorization and land protection choices in GTAP-BIO-ADV and GCAM v5.1. Although we focus on these two models, we posit that land representation choices in all models used for biofuel LUC simulation influence the emission estimates produced by those models and thus should be understood whenever comparing model results.

### 4.1. Land representation in GTAP-BIO-ADV

Most of the models that rely on the GTAP database for some of their data (e.g., MIRAGE-BioF, EPPA, GCAM, GTAP-Dyn), as well as the GTM, on which GTAP’s forestry data are based, provide methods to bring non-commercial land into economic use. GTAP-BIO-ADV does not. Some GTAP modelers have recognized that excluding natural and non-commercial forests will tend to “understate total carbon releases and overstate forest reversion” (Hertel et al., 2010). As shown above, our results are consistent with this claim.

In describing the GTM, Sohngen and colleagues refer to forested land that is too costly to harvest under current prices as “inaccessible”, which contradicts the common usage of this word. For example, Sohngen et al. (1999) write, “There are two sources of new supply in forest management: new harvests in remaining inaccessible natural forests and heavier regeneration investments …”. GTM allows the conversion of “inaccessible” natural forest to commercial use by representing access costs as a barrier that may be overcome when prices rise. This does not occur in GTAP-BIO-ADV, which treats these forest lands as categorically inaccessible at any price, by removing them from consideration by the model’s land substitution equations. Ignoring land deemed inaccessible under current prices tends to underestimate land conversion in response to price changes (Gouel and Hertel, 2006).

Moreover, deeming forest land “inaccessible” based on its proximity to roads based on FAO’s assessment in the year 2000 likely underestimates accessibility today and in the coming decades over the modeled time horizon. For decades, South American, African, and Asian countries have supported roadway development deep into tropical forests in support of expanded logging and agricultural development (Kleinschroth et al., 2019; Laurance et al., 2009). These infrastructure expansions beget additional land clearing and related environmental impacts; 95% of deforestation in the Amazon has occurred within 6 km of roadways or 1 km of waterways (Barber et al., 2014). According to Vilela et al. (2020), if the rate of current agricultural expansion continues in the Amazon, driven by roadway expansion, 40% of Amazonian forest will be cleared by 2050. Several studies have documented the conversion of non-commercial ecosystems to agricultural uses, especially over multi-decadal periods. Across the tropics between 1980 and 2000, over half of new agricultural land replaced intact forests (Gibbs et al., 2010). Potapov et al. (2017) report that the area of intact forest land (i.e., areas with no remotely detected signs of human activity and a minimum area of 500 km^2^) was reduced by 7% (over 900,000 km^2^) from 2001 to 2013, with industrial logging, agricultural expansion, fire, and mining/resource extraction as the primary drivers. Wade et al. (2020) found that even protected areas, in some key geographies, have experienced anthropogenic forest loss for agriculture.

We note that the afforestation and carbon uptake effects seen in GTAP Land Proxy, and their resulting reduction in CI-LUC, are consequences of a land representation that excludes the possibility of bringing non-commercial land into productive use. Our GTAP Land Proxy model results suggest that models of LUC emissions that exclude the conversion of non-commercial land should be expected to produce lower CI-LUC estimates than they would be if this possibility were represented. The conversion of unmanaged land to economic use is one of the key dynamics of relevance to carbon intensity, and therefore an essential element in modeling biofuel-induced LUC.

### 4.2. Land representation in GCAM v5.1

As discussed in Section 1.1.1, GCAM categorizes forest as either commercial or non-commercial. This relatively simple representation glosses over some aspects of actual forestry systems, such as selective or periodic harvests of otherwise non-commercial forests. Pasture in GCAM is similarly either commercial or non-commercial, with no possibility of grazing areas designated non-commercial. Although not quantified here, these simplifications may be worthy of additional investigation.

### 4.3. Conclusion

We find that the representation of land categories and of transitions among these categories strongly influence modeled biofuel CI estimates. If non-commercial land is not represented, all land conversions associated with increased demand for an agricultural product necessarily occur on commercial land. When this conversion reduces the supply of forestry commodities it causes partial replacement of commercial forest to occur elsewhere, offsetting LUC emissions, and resulting in lower overall CI estimates compared to when non-commercial land is represented and available for conversion. Excluding non-commercial land conversion also adds pressure on agricultural- and forestry-commodity prices, resulting in price-induced yield responses and demand destruction that reduce LUC estimates.

Our results also show that replacing the default 90% land protection assumption in GCAM v5.1 with estimates of available land based on suitability and present-day protections results in higher CI-LUC estimates, by virtue of increasing the amount of land that is available for conversion to agricultural use. Based on our review of the underlying data, we believe that using these, or similar, estimates of available land to inform the land protection assumption is an enhancement that could be made in future public releases of GCAM and other models that simulate agricultural expansion.

Supporting information is available online, including a document with addition text, figures, and tables, and in that document, a link to additional code, spreadsheets, and data used to construct the models described herein.

## Supplementary Material

SI

## Footnotes

1 CET: constant elasticity of transformation; CGE: computable general equilibrium; CI: carbon intensity; CI-LUC: carbon intensity of land use change; GCAM: Global Change Analysis Model; GHG: greenhouse gas; GTAP: Global Trade Analysis Project; GTM: Global Timber Model; LUC: land use change; MCS: Monte Carlo Simulation; OTAQ: Office of Transportation and Air Quality (in the U.S. Environmental Protection Agency).
