# Supplementary material for: Choices in land representation materially affect modeled biofuel carbon intensity estimates
Source: J Clean Prod. Author manuscript; Available in PMC 2022 May 25. (PMC9132210; doi:10.1016/j.jclepro.2022.131477)
Supplement: SI [file NIHMS1793721-supplement-SI.pdf]

Choices in land representation materially affect modeled biofuel carbon intensity estimates

Richard J. Plevin, Jason Jones, Page Kyle, Aaron Levy, Michael Shell, Daniel Tanner

Contents: 6 tables and 9 figures

Note: additional supporting information can be downloaded as a zip file from https://dx.doi.org/10.5281/zenodo.5093793. This includes:

- *Spreadsheets with data used to create (i) new geographic regionalization, and (ii) new land representation*
- *Python scripts that extract CSV files from the above and format them as required for the GCAM data system*
- *Modified input files used in the GCAM data system (generated from the spreadsheets and scripts above)*
- *Modified R script to handle new land representation, including files generated by the above*
- *Input files for pygcam to run the scenarios modeled, and to compute carbon intensity*

S1 GCAM-T

GCAM-T 2020.0 (hereafter GCAM-T), is developed through modifications to GCAM v5.1.2 (Calvin, Patel et al. 2019). Some of the modifications developed for GCAM-T have been incorporated into subsequent versions of GCAM. The current release as of this writing is v5.3. Key enhancements compared to GCAM v5.1 include regional agricultural markets and trade, upstream energy and agricultural linkages, disaggregated oil crops, and updated crop yields based on 2015 data. The full GCAM-T release (JGCRI 2020), along with documentation of modifications compared to GCAM v5.1, is free and available at http://dx.doi.org/10.5281/zenodo.4705472.

S1.1 Land Protection

Table S1 shows the fraction of non-commercial land protected in GCAM-T, with area-weighted totals by land category and aggregated region. Unlike the default approach which protects 90% of all unmanaged land categories in all regions, the "land suitability" approach shows substantial heterogeneity across regions and land categories. Notably, no land category in any region is 90% protected; only non-commercial forest and pasture in Canada and Russia are more than 70% protected.

Table S1. Fraction of non-commercial land protected in GCAM-T as a result of land suitability assessment. The column labeled "Total" shows the fraction of non-commercial land protected in each region. The row labeled "Total" shows the overall fraction of land protected in each category, globally. In total, 36% of all non-commercial land is protected globally.

Region	Non-commercial Pasture	Non-commercial Forest	Shrubland	Grassland	Total
Brazil	8%	5%	11%	7%	9%
Canada	74%	87%	69%	44%	72%
China	37%	53%	20%	54%	45%
Europe	16%	21%	14%	11%	15%
India	17%	11%	24%	19%	19%
Indonesia	15%	0%	13%	14%	13%

Mid. East/N. Africa	42%	45%	49%	62%	57%
Oceania	35%	44%	19%	30%	35%
Other Americas	28%	18%	14%	27%	22%
Other Asia	39%	58%	14%	68%	49%
Russia	72%	85%	62%	40%	67%
Sub-Saharan Africa	11%	26%	5%	19%	15%
USA	40%	58%	27%	21%	32%
Total	36%	30%	51%	35%	36%

S1.2 GTAP Land Proxy version of GCAM-T

An outline of the steps involved in producing the GTAP Land Proxy version of the model are presented in the main article. Here we present further details on how the GCAM-T model was modified to produce this new version.

Our intention was to replicate several key features of the GTAP-BIO-ADV land representation:

- The area represented as producing timber is assumed to be all land deemed "accessible forest" by an analysis the Food and Agriculture Organization (FAO 2000).
- To maintain the baseline timber production over this larger area, timber yield is reduced, so that the product of yield and area matches reported timber production.

The regional areal extent for each land category, shown in Table S2 and Figure S1, was read from a GTAP-BIO-ADV data file and copied into an Excel workbook, also available as part of the Supporting Information.

Table S2. GTAP-BIO-ADV land area by region (Mha)

Region	Forestry	Cropland	Livestock	Unmanaged
Brazil	154	63	174	164
Carib. and Cent. America	49	57	84	56
Canada	101	36	20	10
China and Hong Kong	152	144	277	98
East Asia	17	5	77	60
EU27	155	123	60	40
India	18	172	10	28
Japan	18	4	0	1
Malaysia and Indonesia	40	75	2	7
Mid. East and N. Africa	2	54	129	151
Oceania	5	34	251	490
Cent./E. Europe, former USSR	54	110	280	116
Other Europe	30	1	1	1
Rest of S. Asia	8	48	37	44
Rest of S.E. Asia	94	59	5	10
Russia	267	124	79	108
South and other America	107	64	256	208
Sub-Saharan Africa	184	227	732	667

United States	232	165	229	124

The GCAM framework includes a subsystem known as the GCAM Data System through which exogenous model inputs are converted, through a series of steps to produce input files expressed in Extensible Markup Language (XML) that are read by the GCAM executable program. These conversion steps are performed by scripts written in the “R” language.

S1.3 Regional alignment

To mimic the GTAP-BIO-ADV approach, we aggregated GCAM data into a set of regions that could be further aggregated to match the 19 GTAP-BIO-ADV regions. In a few cases, countries in the standard GCAM 32-region aggregation were re-aggregated to allow post-model run aggregation: Argentina and Colombia were moved into the GCAM region “South America (Southern)” and “South America (Northern)”, respectively, and several small (typically island) nations were moved into different aggregate regions.

To create this aggregation in GCAM-T required updating several CSV files in the GCAM Data System. This was accomplished using an Excel Workbook (GCAM-GTAP-regions-v3.xlsx) to compute the values required in worksheets that were extracted by a Python script and written to the required CSV file names.

S1.3.1 Forest and pasture alignment

The extent of commercially active forest and pasture are computed in the GCAM data system by dividing historical production by yield. To approximate the forest and pasture areas represented in the GTAP database, we computed “yield adjustment” factors (in GCAM-GTAP-land-comparison-v9.xlsx) for each GTAP region and applied these to the corresponding regions in the GTAP Land Proxy model.

Where region boundaries matched exactly between the two models (e.g., USA, China, Brazil, Japan, others) we simply applied the yield adjustment to the corresponding region. Where GTAP regions were represented by a group of regions in GTAP Land Proxy, we examined the total forest and pasture area in each region in the group to ensure that the yield scaling would not exhaust the available land. If so, we limited the yield adjustment to use at most 95% of the total forest or pasture and increased the yield adjustment factor in other GCAM regions in that group. We applied the resulting yield adjustments to the modified data system used to generate inputs for the GTAP Land Proxy. We then modified the R script that computes forest and pasture areas to read and apply the scaling factors.

Corresponding changes were made to areal carbon density data to ensure these were consistent with the scaled timber and pasture (feed) yields. We adjusted assumed commercial forest and pasture vegetation carbon densities to conserve the total vegetative carbon stocks of forests and pastures in each land use region of GCAM-T. Because commercial (i.e., logged) and non-commercial forests are assigned different average vegetation carbon densities in GCAM, simply re-assigning a region’s forested lands from "non-commercial" to “commercial” with no further adjustments would cause a reduction in the region’s total forest vegetative carbon stocks. The assumed carbon densities of commercial forests and pastures are thus revised upwards from the values in GCAM-T

Finally, we protected all remaining non-commercial land, including all land designated as shrubland and grassland in GCAM-T, as well as all remaining non-commercial forest and pasture, as none of these land

categories are used by the GTAP-BIO-ADV model. Table S3 shows the managed forest and pasture areas in GCAM, the values from GTAP-BIO-ADV, and the small differences between them.

Based on the areas in **Error! Reference source not found.**, we calculate that the total area of protected land in GTAP Land Proxy (5,832 M ha) represents 65% of the non-commercial land area (less Other Arable) in GCAM-T. That is, if we protected 5,832 Mha of non-commercial land in GCAM-T that would leave 3,210 Mha unprotected non-commercial land less Other Arable (11,007 arable – 5,832 protected – 1,557 commercial – 408 other arable = 3,210 unprotected non-commercial less other arable). Thus, 65% of non-commercial land less other arable would be protected, i.e., 5,832 protected / (5,832 protected + 3,210 non-commercial unprotected less other arable) = 65%.

Table S3. Land area in GTAP-BIO-ADV and GTAP Land Proxy (GCAM) model

Region	GTAP Forest	GCAM Forest	Forest difference	GTAP Pasture	GCAM Pasture	Pasture difference
Brazil	153.5	153.5	0.00	173.7	173.7	0.00
China and Hong Kong	135.1	135.1	0.00	277.0	277.0	0.00
Carib. and Cent. America	49.0	49.0	0.00	83.8	83.8	0.04
Canada	100.5	100.5	0.00	14.1	14.1	0.00
EU27	132.9	133.0	-0.09	60.1	60.1	0.00
East Asia	10.9	10.9	0.00	76.7	76.7	0.00
India	18.1	18.1	0.00	10.1	10.1	0.00
Japan	18.4	18.4	0.00	0.0	0.0	0.00
Mid. East and N. Africa	1.1	1.2	-0.01	129.5	129.5	0.00
Malaysia and Indonesia	39.9	39.9	0.00	2.4	2.4	0.00
Oceania	5.1	5.1	0.00	251.3	251.3	0.00
Cent./E. Europe, former USSR	30.0	29.8	0.21	270.5	270.4	0.02
Other Europe	14.0	14.0	0.00	1.1	1.1	0.00
Rest of S.E. Asia	93.5	93.5	0.00	5.1	5.1	0.00
Rest of S. Asia	7.6	7.6	-0.01	36.4	36.4	0.00
Russia	267.4	267.4	0.00	78.5	78.5	0.00
Sub-Saharan Africa	183.6	183.7	-0.10	731.6	731.7	-0.10
South and other America	107.4	107.4	0.00	254.4	254.4	0.00
United States	211.2	211.2	0.00	229.2	229.2	0.00

S2 Monte Carlo Simulation

S2.1 Parameter distributions

Inputs to GCAM are defined using a hierarchical representation in XML format. The model's input files define tens of thousands of numerical values describing emissions, conversion efficiencies, elasticities, share-weights, logit exponents, and more, with values that can differ by region, sector, subsector, year, and river basin. In the Monte Carlo framework used here—pygcam (Plevin 2020)—a "parameter" is a name associated with a set of input values identified in one of GCAM's input XML files. Related sets of numerical values are identified using the XML Path Language (XPath) and manipulated together as a single named parameter according to a specified distribution. Distributions are applied to all of the values returned, either individually (i.e., independent) or together as a set (i.e., perfectly correlated). In addition, correlations between 0 and 1 can be defined among sets of parameter values. This framework

is general enough to allow the analyst to be as specific or broad as desired in deciding which values to perturb together.

lists the parameters treated stochastically in this analysis, along with the distributions assigned. The parameters fall broadly into three categories: (i) logit exponents that influence the substitutability between different land cover types in response to a biofuel shock, (ii) the soil and biomass carbon density of various land cover types, and (iii) the income and price elasticity of food crops and meat. Allowing for greater income and price elasticity of food demand has the effect of reducing LUC emissions by virtue of reducing consumption of land-intensive food, particularly meat. The input file used by pygcam to drive the simulation (parameters.xml) is provided in as additional on-line supporting information.

Table S4. Parameter distributions used in the Monte Carlo simulations. A value of “multiply” in the Application column indicates that draws from the given distributions were multiplied by the GCAM default values to produce values for each trial. A value of “direct” indicates that values from the distribution were used directly, replacing the default values. Note that more reader-friendly variable names were used in the main text of the paper: “Forest / Grassland / Cropland Competition” (in the main text) = “forest-grass-crop-logit-exp” (in this table); “Soil Carbon Density – Cropland” = “crop-soil-c”; “Crop Competition” = “crop-logit-exp”; “Soil Carbon Density – Cropland” = “grass-soil-c”; “Soil Carbon Density – Other Arable Land” = “other-arable-soil-c”.

Name	Distribution	Application	Description
agro-forest-logit-exp	Triangle(min=0.333, mode=1, max=3.0)	multiply	Logit exponent controlling competition between forest-grass-crop and pasture.
bd-biomassOil-coef	Triangle(min=0.95, mode=1, max=1.05)	multiply	The EJ of biomass oil required to produce an EJ of biodiesel.
corn-etoh-corn-coef	Triangle(min=0.98, mode=1, max=1.02)	multiply	The EJ of corn required to produce an EJ of corn ethanol.
cow-sheepgoat-feed-logit	Triangle(min=0.5, mode=1, max=2.0)	multiply	Logit exponent controlling competition between Beef, Dairy, and SheepGoat, which determines the sharing between Mixed and Pastoral subsectors.
crop-biomass-c	Triangle(min=0.7, mode=1, max=1.3)	multiply	Biomass carbon density of cropland.
crop-logit-exp	Triangle(min=0.333, mode=1, max=3.0)	multiply	Logit exponent controlling competition among crops.
crop-productivity	Triangle(min=0.7, mode=1, max=1.3)	multiply	Annual change in agricultural productivity (yield).
crop-soil-c	Triangle(min=0.7, mode=1, max=1.3)	multiply	Soil carbon density of cropland.
food-crop-price-elast	Triangle(min=-0.2, mode=0.0, max=0.0)	direct	Price elasticity of food crop demand. Standard value is 0 in all regions.
forest-grass-crop-logit-exp	Triangle(min=0.1, mode=1.0, max=3.0)	multiply	Logit exponent controlling competition among forest, grassland, and cropland.
forest-logit-exp	Triangle(min=0.333, mode=1, max=3.0)	multiply	Logit exponent controlling competition between managed and unmanaged forest.
grass-biomass-c	Triangle(min=0.7, mode=1, max=1.3)	multiply	Biomass carbon density of unmanaged grass land.
grass-soil-c	Triangle(min=0.7, mode=1, max=1.3)	multiply	Soil carbon density of unmanaged grass land.
irrig-rainfed-logit-exp	Triangle(min=0.333, mode=1, max=3.0)	multiply	Logit exponent controlling competition between irrigated and rainfed land.
meat-demand-share-logit	Triangle(min=-2.0, mode=0.0, max=0.0)	direct	Logit exponent controlling shifting between types of meat. Standard value is 0 in all regions.
meat-price-elast	Triangle(min=0.5, mode=1, max=2.0)	multiply	Price elasticity of meat demand.

mgd-forest-biomass-c	Triangle(min=0.7, mode=1, max=1.3)	multiply	Biomass carbon density of managed forest land.
mgd-forest-soil-c	Triangle(min=0.7, mode=1, max=1.3)	multiply	Soil carbon density of managed forest land.
mgd-pasture-biomass-c	Triangle(min=0.7, mode=1, max=1.3)	multiply	Biomass carbon density of managed pasture.
mgd-pasture-soil-c-linked	Linked(parameter=grass-soil-c)	multiply	Soil carbon density of managed pasture.
mgmt-level-logit-exp	Triangle(min=0.333, mode=1, max=3.0)	multiply	Logit exponent controlling competition between high and low crop management levels.
N-fertilizer-rate	Triangle(min=0.7, mode=1, max=1.3)	multiply	Quantity of N fertilizer required per mass of crop harvested.
n2o-emissions	Triangle(min=0.5, mode=1, max=2.0)	multiply	N2O emissions intensity of agricultural production.
ng-upstream-ch4	Uniform(min=0.9, max=1.3)	multiply	CH4 emissions upstream from natural gas production processes and transport.
other-arable-biomass-c	Triangle(min=0.7, mode=1, max=1.3)	multiply	Biomass carbon density of "other arable" land.
other-arable-soil-c	Triangle(min=0.7, mode=1, max=1.3)	multiply	Soil carbon density of "other arable" land.
pass-road-ldv-4W-logit-exp	Triangle(min=0.5, mode=1, max=2.0)	multiply	Logit exponent controlling substitution among Compact Car, Midsize Car, Large Car, Light Truck and SUV.
pass-road-ldv-4W-vehicle-logit-exp	Triangle(min=0.5, mode=1, max=2.0)	multiply	Logit exponent controlling substitution among 4WD vehicle fuel technology options include BEV, FCEV, Hybrid liquids, Liquids, and NG.
pass-road-ldv-logit-exp	Triangle(min=0.5, mode=1, max=2.0)	multiply	Logit exponent controlling substitution between 2- and 4-wheel light-duty vehicles.
pasture-logit-exp	Triangle(min=0.333, mode=1, max=3.0)	multiply	Logit exponent controlling competition between managed and unmanaged pasture.
population-factor	Triangle(min=0.0, mode=0.5, max=1.0)	direct	Defines a path between the lower and higher bounds of the UNDP 95% confidence interval around population projections.
regional-crop-logit-exp	Triangle(min=0.333, mode=1, max=3.0)	multiply	Logit exponent controlling competition between imports and domestic ag products.
shrub-biomass-c	Triangle(min=0.7, mode=1, max=1.3)	multiply	Biomass carbon density of shrubland.
shrub-soil-c	Triangle(min=0.7, mode=1, max=1.3)	multiply	Soil carbon density of shrubland.
traded-crop-logit-exp	Triangle(min=0.333, mode=1, max=3.0)	multiply	Logit exponent controlling shares of regions supplying the global market through exports.
unmgd-forest-biomass-c	Triangle(min=0.7, mode=1, max=1.3)	multiply	Biomass carbon density of unmanaged forest land.
unmgd-forest-soil-c	Triangle(min=0.7, mode=1, max=1.3)	multiply	Soil carbon density of unmanaged forest land.
unmgd-pasture-biomass-c-linked	Linked(parameter=grass-biomass-c)	multiply	Biomass carbon density of unmanaged pasture (linked with grass-biomass-c).
unmgd-pasture-soil-c-linked	Linked(parameter=grass-soil-c)	multiply	Soil carbon density of unmanaged pasture (linked with grass-soil-c).
water-wd-price	Triangle(min=0.333, mode=1, max=3.0)	multiply	The price of withdrawn water.

Note that some of the parameters listed in Table S4 were included in the MCS for use in other analyses and have little, if any, influence on CI-LUC. These include parameters related to emissions of N_2O and CH_4, and those related to vehicle choice.

Figure S1. Baseline land allocation in GTAP-BIO-ADV by land category. The "Crops" category includes active cropland and cropland-pasture. Unmanaged land ("Unmngland") includes "inaccessible" forest, urban land, ice, and tundra, and cannot be brought into economic use in GTAP-BIO-ADV.

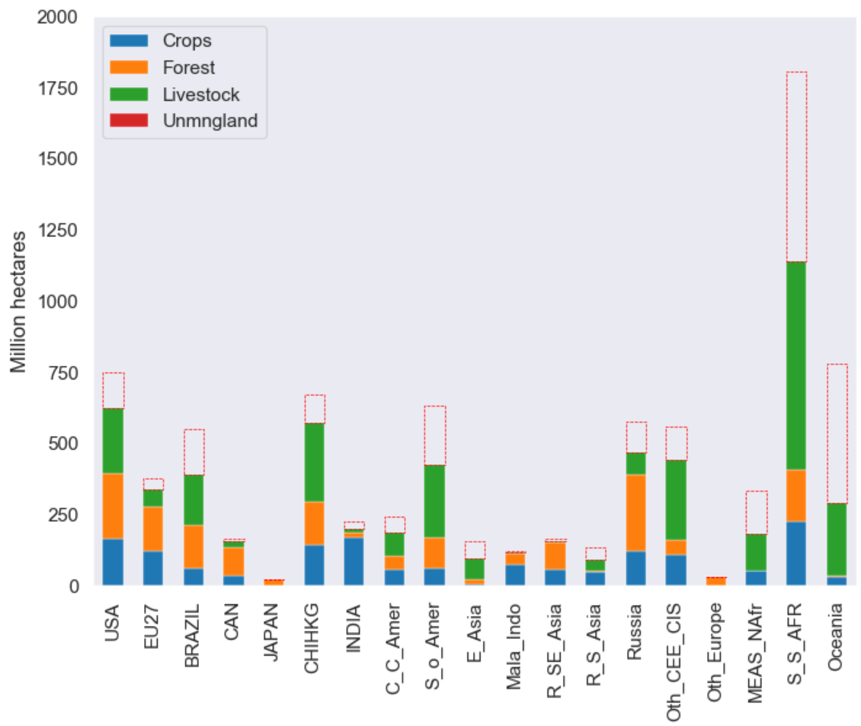

Figure S2. Corn ethanol shock – the additional ethanol production required in the policy scenarios above the baseline AEO 2018 projections.

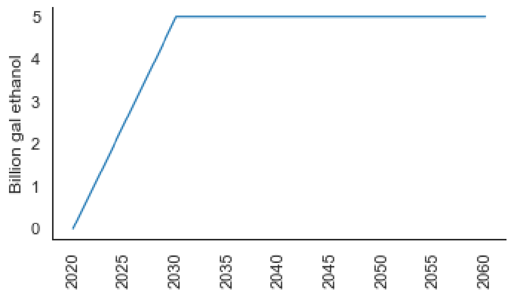

S3 Scenario design

With each version of the model, we created a reference scenario in which domestic U.S. biofuel consumption volumes are set exogenously to 2015 historical levels for all modeled years after the 2010 base year. We compared this reference scenario to a scenario with an additional 5 billion gallons of corn ethanol production annually in the U.S. from 2030 through 2060, and a linear ramp-up to the additional 5 billion gallons from zero additional gallons in 2020 to 5 billion additional gallons in 2030 (see Figure S2).

S4 Land competition and substitution

In addition to the choice of land categories, models represent differently the dynamics of LUC—in particular, the competition among and allowable transitions between land uses. There are two fundamental ways that models may protect different types of land transitioning across land uses… A key question in estimating land use change emissions is how models account for the conversion of non-commercial (sometimes called "unmanaged") land into commercial use. Models that represent non-commercial land differ in how they model conversion of this land for productive use.

GLOBIOM, a global partial equilibrium model, uses constrained optimization with conversion costs in each region to convert natural vegetation, grassland, and non-commercial forest to agricultural or timber production. GCAM represents land in a multi-level nesting structure; within each nest, exogenous logit parameters determine the level of substitutability. In this framework, LUC is driven by the endogenous relative profitability of competing land uses subject to this estimate of substitutability.

Computable general equilibrium (CGE) models (e.g., ADAGE, EPPA, GTAP-BIO, GTAP-Dyn, MIRAGE) commonly use a constant elasticity of transformation (CET) function (Hertel, Rose et al. 2009) that adjusts the land supply based on the share of total returns for each land type and the elasticity of transformation (Babcock and Carriquiry 2010). Both the CET and logit approaches treat conversion as symmetric between land uses, e.g., cropland can be converted to forest as readily as forest to cropland. Similarly, both approaches usually employ a nesting strategy to differentiate substitutability among and within broader land categories (see Figure S4 and Figure S5, below.)

The GTAP-BIO-ADV model includes a crop category in the US, Brazil, and Canada called "cropland-pasture", which is defined as land that rotates between crop production and grazing. In GTAP-BIO-ADV, there is a substantial quantity of this land, particularly in the US, and it is the source of most of the new cropland required in response to a corn ethanol shock, as shown in Figure S3.

The GCAM model does not explicitly represent cropland-pasture, though it does include an unmanaged category of land called simply "other arable land", which includes fallow land and also represents differences in land area estimates between USDA and other data sources.

GTAP-BIO-ADV represents cropland-pasture as a type of crop (Figure S4) facilitating the transition of cropland-pasture to cropland; the transition of forest and pasture to cropland is more difficult. In GCAM, (Figure S5), non-commercial forest and pasture and grassland, which have relatively low economic value, are most readily converted to cropland.

In GTAP-BIO-ADV, forestry, pasture, and cropland compete on an equal basis at the top level of the nesting structure. In GCAM, non-commercial land classes compete with their commercial counterparts, and forest competes with cropland and the combined grassland/shrubland nest. Competition with pasture occurs at a higher level in the nesting structure and is thus somewhat more resistant to conversion.

S5 Land use change emissions

Figure S6 compares the LUC emissions projected by the GCAM-T and GTAP Land Proxy models. In both models, most emissions occur in the U.S. However, the GTAP Land Proxy model shows ongoing, though

declining, emissions throughout the modeled time horizon, while emissions in the GCAM-T model decline sharply after 2030 and then taper off quickly by about 2045.

Figure S7, showing land allocations over time, helps explain the difference in emission profiles. In the GCAM-T case, new cropland required to meet the ethanol shock is acquired primarily by converting non-commercial forest, pasture, and other non-commercial land. (In GCAM's carbon accounting system, biomass loss from land conversion is assumed to happen in the year of conversion.) Thereafter, changes are slowly reversed, presumably as yield growth outpaces population growth, reducing overall land requirements. The lower, but continued emissions post-2030 would be from soil carbon, which is modeled in GCAM with an exponential decay curve.

Figure S3. Land use change in GTAP-BIO for expansion of corn ethanol. The values presented here are for the "Corn ethanol 2013" GTAP results provided in the CCLUB_2019 model, distributed with GREET_2019.

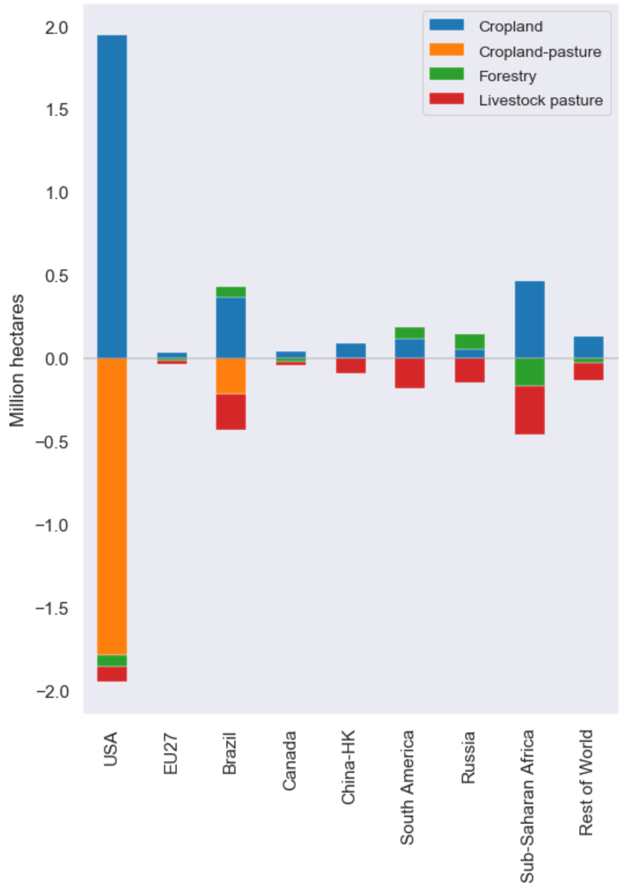

In the GTAP Land Proxy model, the baseline (Figure S7a, third column) shows steady growth in commercial forest (green line) and loss of commercial pasture (brown line). These baseline trends are both reversed in the change (Figure S7b): commercial forest declines, then holds at about 0.5 Mha less than in the baseline, while commercial pasture declines initially, then rises slightly over time. Cropland grows, and then declines as yields increase post-2030, with the corresponding increases in "other arable" and commercial pasture. These changes all result in an accumulation of soil carbon, but also less forest growth, so less accumulation of tree biomass over time. The difference is thus largely a result of

the asymmetry of biomass loss and recovery, where loss occurs in one year and recovery occurs over years for grass and shrubland and decades for forests.

Figure S4. GTAP-BIO-ADV land nest. Cropland-Pasture is represented as a crop, though the land can be converted to Pasture.

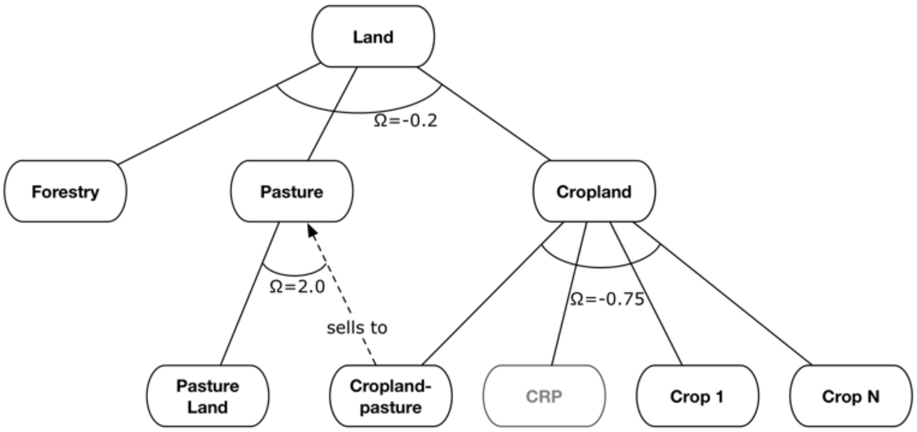

Figure S5. GCAM-T land nest. Commercial land use categories are shown in blue; non-commercial land-cover categories are shown in green.

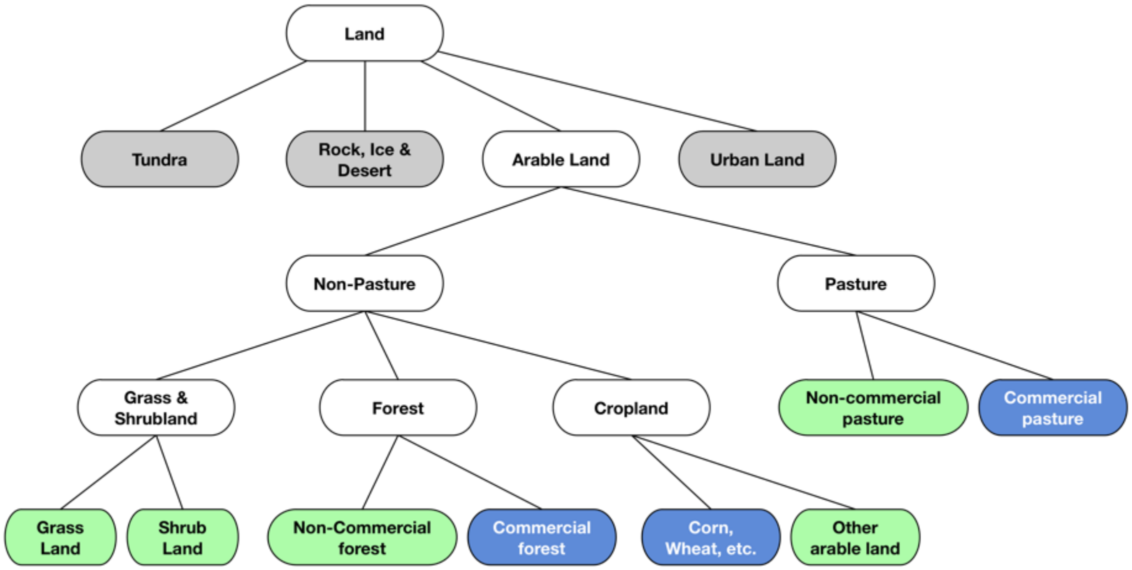

Figure S6. Land use change emissions over time for GCAM-T and GTAP Land Proxy models.

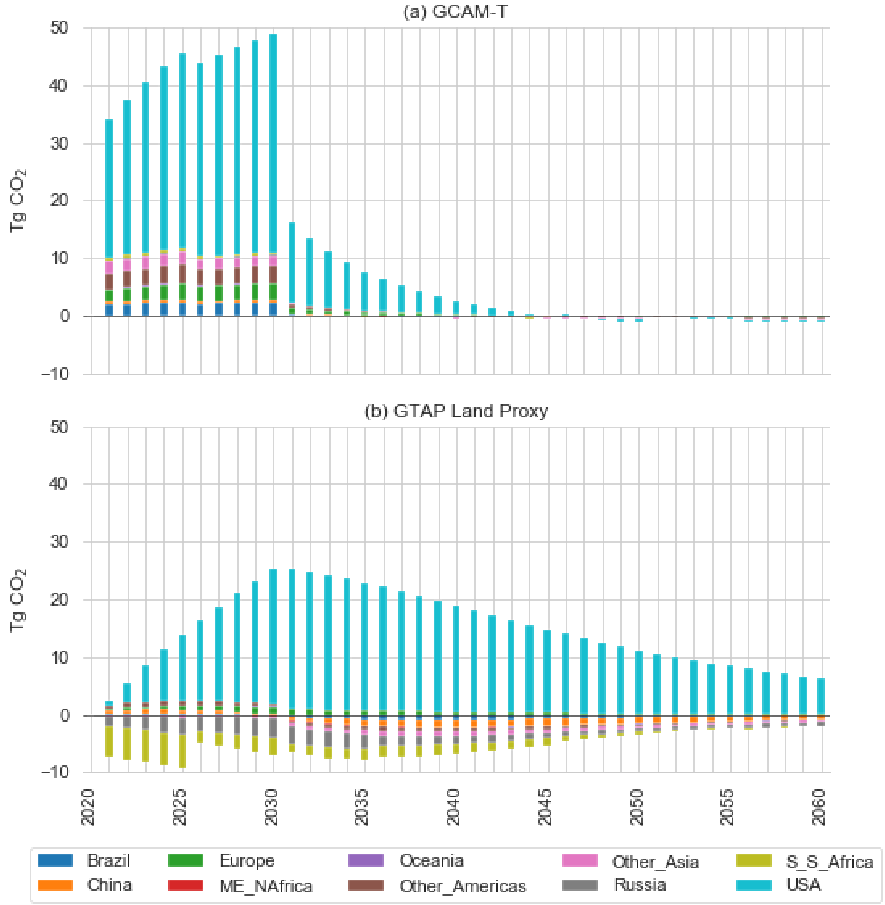

Figure S7. Land allocation in the three model versions: (a) baseline allocation, and (b) changes from baseline resulting from increased corn ethanol production in the U.S.

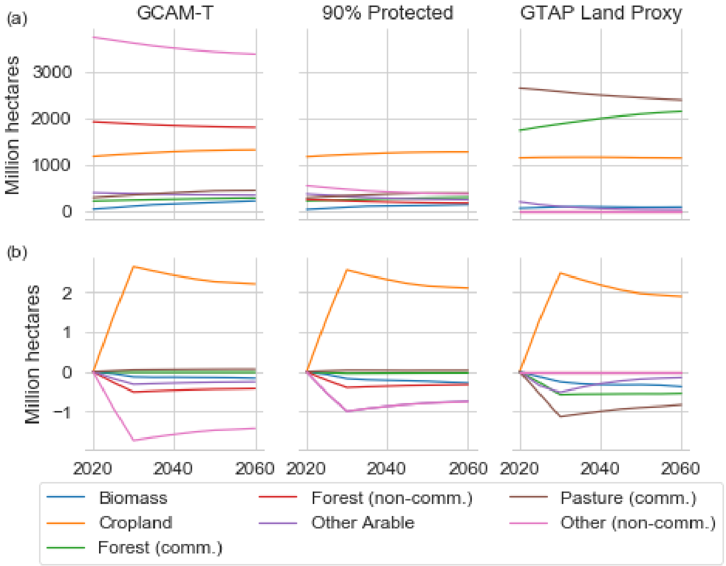

S6 Effect of time horizon on CI-LUC

As noted in the main text, different jurisdictions have chosen different time horizons for evaluating CI-LUC, with US regulators using 30 years and EU regulators using 20 years. Figure S8 shows the effect of different choices of end year time horizon used to account for changes in GHG emissions and U.S. corn ethanol production. Accounting starts in 2020 in all cases.

Since most of the emissions (the numerator of CI-LUC) occur by 2030, longer time periods mainly increase the total corn ethanol production (the denominator of CI-LUC) resulting in lower CI-LUC values.

Figure S8. CI-LUC as a function of the end year of the accounting time horizon, which starts in 2020.

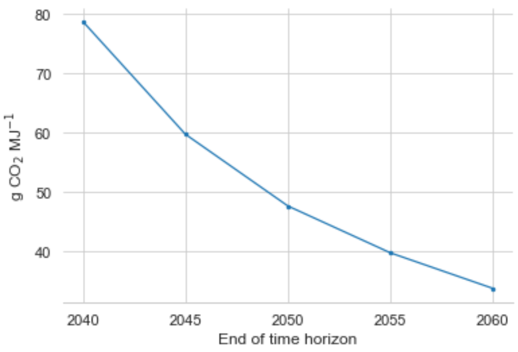

S7 Additional tables and figures

Table S5. Global land areas (Mha) in the three model versions used for Figure 4 in main text.

	GTAP Land Proxy	GCAM-T	90% Protected
Protected Forest	1,423	844	2,527
Forest (unmanaged)	0	1,964	281
Forest (managed)	1,579	194	194
Protected Pasture	617	1,077	2,754
Pasture (unmanaged)	0	1,984	306
Pasture (grazed)	2,685	241	241
Protected Shrubland	1,177	595	1,059
Shrubland	0	581	118
Protected Grassland	1,996	696	1,797
Grassland	0	1,301	200
Cropland	1,123	1,121	1,121
Other Arable	406	408	408
Non-arable	2,162	2,162	2,162
Total	13,169	13,169	13,169
Arable	11,007	11,007	11,007
Protected	5,213	3,212	8,138
Commercial	5,388	1,557	1,557
Unprotected non-commercial	406	6,238	1,313
Unprotected non-commercial (less other arable)	0	5,830	904
% Non-commercial (less other arable) protected	100%	36%	90%

Table S6. Arable land categorization in several models used to estimate biofuel-induced land use change.

Model	Arable Land Categories	Geopolitical Regions	Crops
ADAGE[1]	Cropland, pasture, commercial forest, non-commercial forest, natural grassland, other land	8	10
EPPA[1]	Cropland, pasture, commercial forest, natural forest, natural grassland	18	7
GCAM[1]	Cropland, commercial pasture and forest, non-commercial pasture and forest, shrubland, grassland, “protected” non-commercial land	32	20
GLOBIOM[2]	Cropland, other agricultural land, grassland, commercial and non-commercial forest, wetlands,[7] other natural land	57	18 (27 in the EU)
GTAP-Dyn[3]	Cropland, livestock pasture, forestry land, non-commercial forest	19	7
GTAP-BIO-ADV[4]	Cropland (including cropland-pasture), livestock pasture, “accessible” forestry land	19	10
MIRAGE-BioF[5]	Arable land, meadows and permanent pasture, permanent crops, commercial and non-commercial forest	11	11
FAPRI-CARD[6]	Cropland; pasture only in Brazil[8]	48 / 53[9]	10

Table S6 notes: 1. Calvin, Beach et al. (2016), 2. Valin, Peters et al. (2015), 3. Golub, Hertel et al. (2008), 4. Taheripour and Tyner (2014), 5. Laborde and Valin (2012), 6. USEPA (2010). 7. Only GLOBIOM represents wetlands as a type of arable land, through a sub-category of wetlands, tropical peat swamps, will be included in an upcoming version of GCAM-T. 8. A GHG sub-model uses the cropland and pasture acreage outputs from the FAPRI-CARD model in relation to the existing proportion of grassland and forestland in any grid-cell to determine the impact on these LUC categories. 9. Carriquiry, Elobeid et al. (2019) have developed subregions for Brazil within FAPRI-CARD.

Figure S9. CI-LUC by fraction of non-commercial land protected.

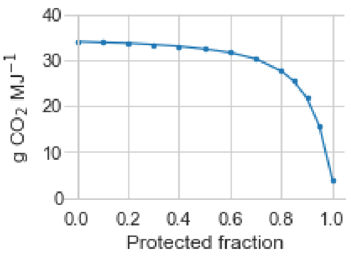

S8 References

Babcock, B. A. and M. Carriquiry (2010). An Exploration of Certain Aspects of CARB’s Approach to Modeling Indirect Land Use from Expanded Biodiesel Production. Ames, IA, Center for Agricultural and Rural Development, Iowa State University: 41.

Calvin, K., P. Patel, L. Clarke, G. Asrar, B. Bond-Lamberty, R. Y. Cui, A. Di Vittorio, K. Dorheim, J. Edmonds, C. Hartin, M. Hejazi, R. Horowitz, G. Iyer, P. Kyle, S. Kim, R. Link, H. McJeon, S. J. Smith, A. Snyder, S.

Waldhoff and M. Wise (2019). "GCAM v5.1: representing the linkages between energy, water, land, climate, and economic systems." Geosci. Model Dev. **12**(2): 677-698.
Calvin, K. V., R. Beach, A. Gurgel, M. Labriet and A. M. Loboguerrero Rodriguez (2016). "Agriculture, forestry, and other land-use emissions in Latin America." Energy Economics **56**: 615-624.
Carriquiry, M., A. Elobeid, J. Dumortier and R. Goodrich (2019). "Incorporating Sub-National Brazilian Agricultural Production and Land-Use into U.S. Biofuel Policy Evaluation." Applied Economic Perspectives and Policy **42**(3): 497-523.
FAO (2000). Global Forest Resource Assessment, Food and Agriculture Organization of the United Nations.
Golub, A., T. W. Hertel and B. Sohngen (2008). "Land Use Modeling in Recursively-Dynamic GTAP Framework (GTAP Working Paper No. 48)."
Hertel, T. W., S. Rose and S. Richard (2009). Land use in computable general equilibrium models: an overview. Economic analysis of land use in global climate change policy, Routledge**:** 23-50.
JGCRI (2020). GCAM-T version 2020.0.
Laborde, D. and H. Valin (2012). "Modeling land-use changes in a global CGE: assessing the EU biofuel mandates with the MIRAGE-BioF model." Climate Change Economics **03**(03): 1250017.
Plevin, R. J. (2020). "Pygcam." from https://pygcam.readthedocs.io.
Taheripour, F. and W. E. Tyner (2014). "Corn oil biofuel land use change emission impacts: sharing emission savings between ethanol and biodiesel." Biofuels **5**(4): 353-364.
USEPA (2010). Renewable Fuel Standard Program (RFS2) Regulatory Impact Analysis. Washington, DC, US Environmental Protection Agency**:** 1120.
Valin, H., D. Peters, M. van den Berg, S. Frank, P. Havlik, N. Forsell, C. Hamelinck, J. Pirker, A. Mosnier, J. Balkovic, E. Schmid, M. Dürauer and F. di Fulvio (2015). The land use change impact of biofuels consumed in the EU: Quantification of area and greenhouse gas impacts, IIASA, E4Tech, and Ecofys.
